# Jaundice revisited: recent advances in the diagnosis and treatment of inherited cholestatic liver diseases

**DOI:** 10.1186/s12929-018-0475-8

**Published:** 2018-10-26

**Authors:** Huey-Ling Chen, Shang-Hsin Wu, Shu-Hao Hsu, Bang-Yu Liou, Hui-Ling Chen, Mei-Hwei Chang

**Affiliations:** 1 Departments of Pediatrics, National Taiwan University College of Medicine and Children’s Hospital, 17F, No. 8, Chung Shan S. Rd, Taipei, 100 Taiwan; 2 Department of Medical Education and Bioethics, National Taiwan University College of Medicine, No. 1, Jen Ai Rd Section 1, Taipei, 100 Taiwan; 3 Hepatitis Research Center, National Taiwan University Hospital, Changde St. No.1, Zhongzhen Dist., Taipei 100, Taiwan; 4 Graduate Institute of Clinical Medicine, National Taiwan University College of Medicine, No. 7 Chung Shan S. Rd, Taipei 100, Taiwan; 5 Graduate Institute of Anatomy and Cell Biology, Nationatl Taiwan University College of Medicine, No. 1 Jen Ai Rd Section 1, Taipei 100, Taiwan

**Keywords:** Cholestasis, Genetic liver disease, Pediatric, Progressive familial intrahepatic cholestasis, Next generation sequencing, Bile acids

## Abstract

**Background:**

Jaundice is a common symptom of inherited or acquired liver diseases or a manifestation of diseases involving red blood cell metabolism. Recent progress has elucidated the molecular mechanisms of bile metabolism, hepatocellular transport, bile ductular development, intestinal bile salt reabsorption, and the regulation of bile acids homeostasis.

**Main body:**

The major genetic diseases causing jaundice involve disturbances of bile flow. The insufficiency of bile salts in the intestines leads to fat malabsorption and fat-soluble vitamin deficiencies. Accumulation of excessive bile acids and aberrant metabolites results in hepatocellular injury and biliary cirrhosis. Progressive familial intrahepatic cholestasis (PFIC) is the prototype of genetic liver diseases manifesting jaundice in early childhood, progressive liver fibrosis/cirrhosis, and failure to thrive. The first three types of PFICs identified (PFIC1, PFIC2, and PFIC3) represent defects in FIC1 (*ATP8B1*), BSEP (*ABCB11*), or MDR3 (*ABCB4*). In the last 5 years, new genetic disorders, such as TJP2, FXR, and MYO5B defects, have been demonstrated to cause a similar PFIC phenotype. Inborn errors of bile acid metabolism also cause progressive cholestatic liver injuries. Prompt differential diagnosis is important because oral primary bile acid replacement may effectively reverse liver failure and restore liver functions. DCDC2 is a newly identified genetic disorder causing neonatal sclerosing cholangitis. Other cholestatic genetic disorders may have extra-hepatic manifestations, such as developmental disorders causing ductal plate malformation (Alagille syndrome, polycystic liver/kidney diseases), mitochondrial hepatopathy, and endocrine or chromosomal disorders. The diagnosis of genetic liver diseases has evolved from direct sequencing of a single gene to panel-based next generation sequencing. Whole exome sequencing and whole genome sequencing have been actively investigated in research and clinical studies. Current treatment modalities include medical treatment (ursodeoxycholic acid, cholic acid or chenodeoxycholic acid), surgery (partial biliary diversion and liver transplantation), symptomatic treatment for pruritus, and nutritional therapy. New drug development based on gene-specific treatments, such as apical sodium-dependent bile acid transporter (ASBT) inhibitor, for BSEP defects are underway.

**Short conclusion:**

Understanding the complex pathways of jaundice and cholestasis not only enhance insights into liver pathophysiology but also elucidate many causes of genetic liver diseases and promote the development of novel treatments.

## Background

Jaundice is a common symptom of inherited or acquired liver diseases of various causes. The underlying biochemical disturbance of jaundice is defined by direct or indirect hyperbilirubinemia. These two categories may represent different mechanisms causing jaundice. Indirect hyperbilirubinemia typically results from increased red blood cell turnover, increased bilirubin loading, or disturbances in hepatocellular update and bilirubin conjugation. Direct hyperbilirubinemia, typically defined as a direct/total bilirubin ratio of more than 15–20%, or a direct bilirubin level above 1.0 mg/dL, is collectively defined as cholestasis.

Recent progress in the past two decades has largely elucidated the molecular mechanisms underlying bile metabolism (including bilirubin, bile acids, cholesterol, phospholipid, and xenobiotics metabolism), hepatocellular transport (including uptake from sinusoidal blood and export to the canaliculus and bile ducts), bile ductular development, the intestinal reabsorption of bile salts, and the regulation of bile acids and cholesterol homeostasis. The understanding of these complex pathways not only provides insights into liver physiology but also elucidates many causes of genetic liver disease and facilitates the development of novel treatments. This review will focus mainly at hepatobiliary causes of jaundice and inherited cholestasis.

## Main text

### The composition and function of bile

The hepatobiliary system comprises the liver, bile duct and gall bladder. Bile is synthesized and secreted by polarized hepatocytes into bile-canaliculi, flows through bile ducts, stored in the gall bladder and is finally drained into the duodenum. The main physiological function of bile is to emulsify the lipid content of food, and this lipid emulsion facilitates lipid digestion and the absorption of lipid-soluble substances. Additionally, bile secretion is an important route to regulate cholesterol homeostasis, hemoglobin catabolism, and the elimination of drugs or drug metabolites [1].

Bile is a yellow-to-greenish amalgam of water, bile acids, ions, phospholipids (phosphatidylcholine), cholesterol, bilirubin, proteins (such as glutathione and peptides) and the other xenobiotics [1]. The yellow-to-greenish color of bile is caused by bilirubin and its derivative, which are also the origin of stool color. Bilirubin is the end catabolite of hemoglobin and other heme-containing proteins, such as myoglobin. The heme molecule is oxidized to biliverdin in hepatocytes and then reduced to unconjugated bilirubin. Unconjugated bilirubin is conjugated with one to two molecules of glucuronic acid via Uridine 5'-diphospho-glucuronosyltransferase 1A1 (UGT1A1). Bilirubin conjugation increases water solubility and reduces cytotoxicity of bilirubin. Hepatic and intestinal UGT1A1 are functionally reduced in neonatal stages, and hence, unconjugated hyperbilirubinemia is commonly found in human neonates [2]. Conjugated bilirubin, or direct bilirubin, is the major form of bilirubin in bile and is eliminated in stool. Jaundice, a yellowish pigmentation of the skin and sclera, is caused by the disrupted excretion of bilirubin and biliverdin. Interestingly, some studies involving neonates or adults have shown that hyperbilirubinemia is protective against diseases, including metabolic syndrome and asthma, [2, 3] suggesting that bilirubin may play a role as an antioxidant [4].

Bile acids are colorless and are the most abundant organic components of bile. Bile acids, a group of detergent-like molecules, are synthesized from cholesterol and are typically associated with sodium or potassium ions in the form of bile salts. Bile salts mediate lipid emulsion and act as signaling molecules to regulate gene expression [5–7]. Phospholipids and cholesterol, the second and third most abundant organic components of bile, protect against injury of the biliary epithelium from bile acids [1].

### Biosynthesis and enterohepatic circulation of bile acids

**Fig. 1:**
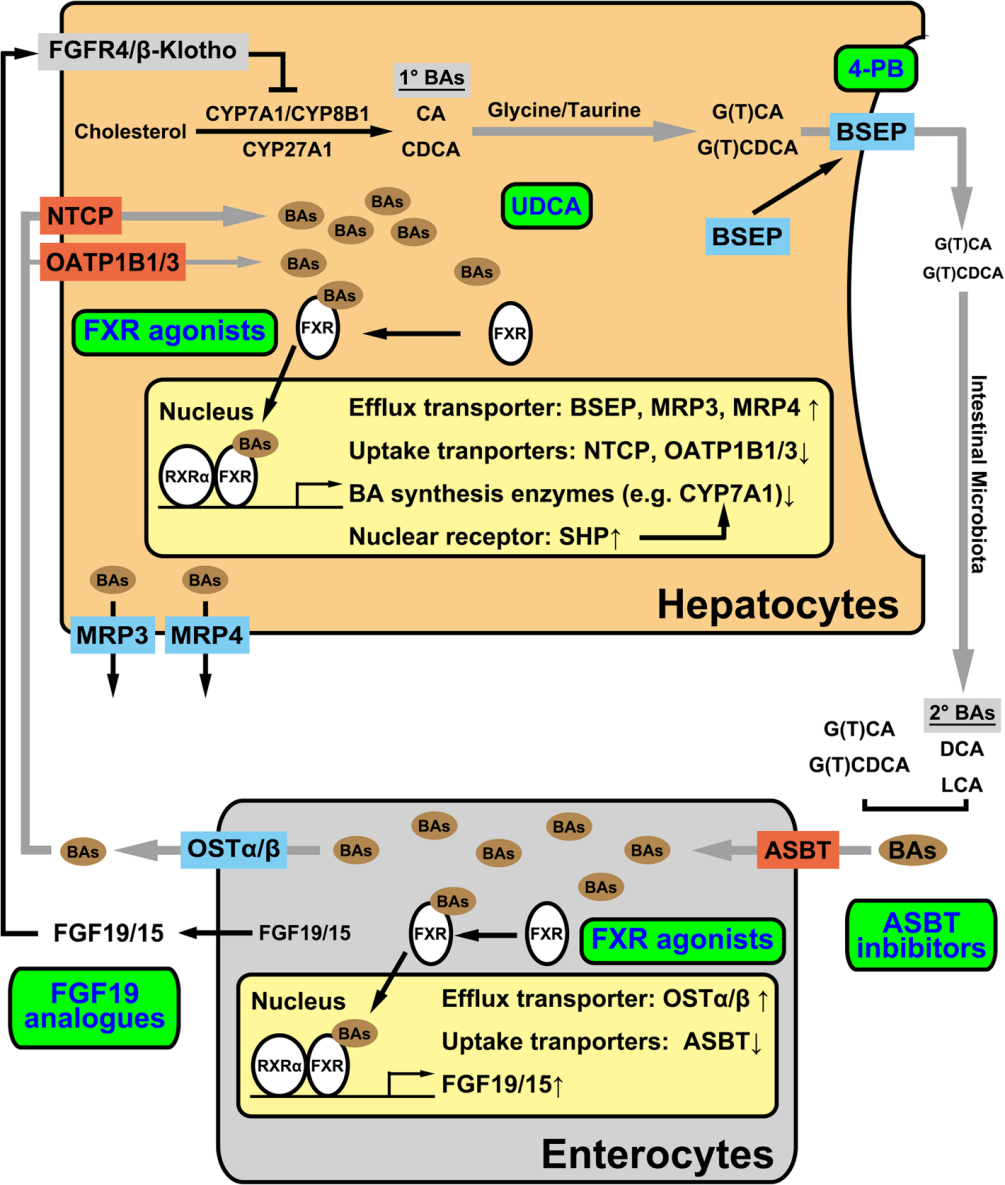
The enterohepatic circulation, homeostasis of bile acids and treatment targets for cholestasis. The grey arrows indicate the route of enterohepatic circulation of bile acids. Bile acids are synthesized from cholesterol in hepatocytes to generate the primary bile acids CA and CDCA. After conjugation with glycine or taurine, bile acids (BAs) are transported from hepatocytes into the bile canaliculi via BSEP. Intestinal microbiota converts primary bile acids into the secondary bile acids DCA and LCA. Most of BAs reabsorbed by the enterocytes through ASBT in the apical membrane and then delivered into the portal circulation system via BA efflux transporter OSTα/β in the basolateral membrane. BAs are re-absorbed into hepatocytes. Hepatocytes secrete these BAs along with the de novo synthesized bile acids enter the next cycle. Bile acids also play roles in signaling to regulate the homeostasis of bile acids. The nuclear receptor FXR is the bile acid receptor to regulate bile acid homeostasis at the synthesis and the elimination levels, acting in the hepatocytes and enterocytes. The figure also shows different therapeutic targets at hepatocellular transport or enterohepatic circulations. 1°BAs, primary bile acids; 2°BAs, secondary bile acids; 4-PB, 4-phenylbutyrate; ASBT, apical sodium dependent bile acid transporter; BAs, bile acids; BSEP, bile salt export pump; CA, cholic acid; CDCD, chenodeoxy cholic acid; DCA, deoxycholic acid; FGFR4, fibroblast growth factor receptor 4; FXR, farnesoid X receptor; G(T)CA, glyco- or tauro-cholic acid; G(T)CDCA, glyco- or tauro-chenodeoxy cholic acid; LCA, lithocholic acid; MRP3, multidrug resistance-associated protein 3; MRP4, multidrug resistance-associated protein 4; NTCP, sodium/taurocholate co-transporting polypeptide; OATP1B1/3, organic-anion-transporting polypeptide 1B1 and 1B3; OSTα/β, organic solute transporter-α/β; RXRα, retinoid X receptor α; SHP, small heterodimer partner; UDCA, ursodeoxycholic acid

Bile acids can be synthesized from cholesterol via two pathways in hepatocytes to generate two primary bile acids, cholic acid (CA) and chenodeoxycholic acid (CDCA), through cytochrome P450 (CYP) enzymes, including CYP7A1, CYP8B1, and CYP27A1. Primary bile acids are conjugated with glycine or taurine (glyco- or tauro-conjugated CA and CDCA), with increased solubility and reduced cytotoxicity. In the intestines, gut-resident microbiota deconjugate bile salts to generate the secondary bile acids, deoxycholic acid (DCA) and lithocholic acid (LCA) [8, 9]. In human livers, de novo synthesized bile salts are 500–600 mg daily [10]. More than 90% of bile acids are reabsorbed at the distal ileum and transported back to the liver through circulation systems for the next cycle, called the enterohepatic circulation. Bile salts cycle 6- to 10-times daily. The total amount of bile salt in the body is called bile acid pool, which is approximately 2–3 g. In contrast to bile acids, only trace amounts of conjugated bilirubin will enter the enterohepatic circulation. The blockage of enterohepatic circulation to enhance bile salt elimination has been applied in surgical and medical treatments for cholestasis (Fig. 1).

In human fetuses after 22 and 26 weeks of gestation, taurine-conjugated di-hydroxyl bile acids can be detected in the gallbladder. After 28 weeks, small amounts of glycine conjugates are synthesized. In postnatal stages, the ratio of CA to CDCA declines from 2.5 to 1.2 [11]. Infant livers are under development, have a small bile acid pool, and have a limited capacity for bile excretion and reabsorption. Therefore, neonates and infants, particularly premature infants, are prone to cholestasis caused by various insults, such as ischemia, drugs, infection, or parenteral nutrition.

### Hepatocellular transporters mediating bile flow (Fig. 2)

Bile flow is generated by osmotic forces associated with the amount of bile salts secreted into bile canaliculi. Bile secretion from hepatocytes is mediated by a group of transport proteins, particularly ATP-binding cassette (ABC) containing proteins. The bile salt export pump (BSEP encoded by *ABCB11*) is the pivotal transporter mediating bile acid transport into bile canaliculi. BSEP is exclusively expressed in the apical/canalicular membrane of hepatocytes. After secreted into the small intestine, bile salts are absorbed into intestinal cells via the apical sodium-dependent bile acid transporter (ASBT encoded by *SLC10A2*) and then secreted into the circulation system through the basolateral heterodimeric transporter OSTα-OSTβ (encoded by *OSTA* and *OSTB*, respectively) [12–14].

**Fig. 2:**
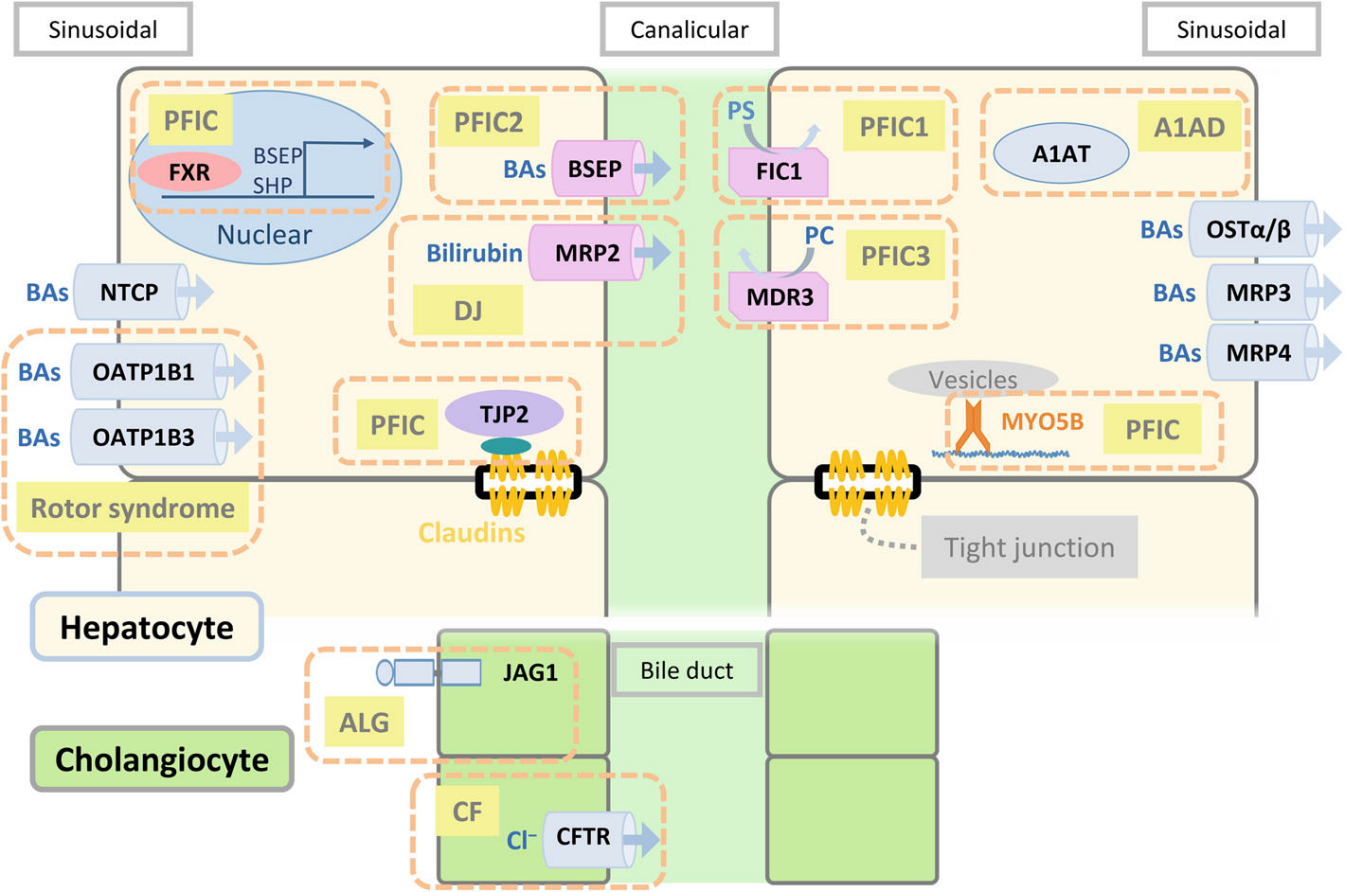
Hepatocellular transporters, enzymes, and regulators involving in bile transport, metabolism, and secretion. A1AD, alpha-1 antitrypsin deficiency; A1AT, alpha-1 antitrypsin; ALG, Alagille syndrome; BAs, bile acids; BSEP, bile salt export pump; Canalicular, canalicular membrane; CF, cystic fibrosis; CFTR, cystic fibrosis transmembrane conductance regulator; DJ, Dubin-Johnson syndrome; FIC1, familial intrahepatic cholestasis 1; FXR, farnesoid X receptor; JAG1, jagged 1; MDR3, multidrug resistance protein 3; MRP2, multidrug resistance-associated protein 2; MRP3, multidrug resistance-associated protein 3; MRP4, multidrug resistance-associated protein 4; MYO5B, myosin VB; NTCP, sodium/taurocholate co-transporting polypeptide; OATP1B1, organic-anion-transporting polypeptide 1B1; OATP1B3, organic-anion-transporting polypeptide 1B3; OSTα/β, organic solute transporter-α/β; PC, phosphatidylcholine; PFIC, progressive familial intrahepatic cholestasis; PS, phosphatidylserine; Sinusoidal, sinusoidal membrane; SHP, small heterodimer partner; TJP2, tight junction protein 2

The basolateral/sinusoidal membrane of hepatocytes contains several bile acid transporters to absorb bile acids from sinusoidal blood, including Na^+^-taurocholate co-transporting polypeptide NTCP (encoded by *SLC10A1*), OATP1B1 and OATP1B3 (encoded by *SLCO1B1* and *SLCO1B3*, respectively) [12, 15]. OATP1B1 and OATP1B3 also function in the uptake of bilirubin into hepatocytes [16]. Conjugated bilirubin and organic anions are transported via canalicular multidrug resistance-associated protein 2 MRP2 (encoded by *ABCC2*) and, to a lesser extent, via ABCG2 into bile. Under physiological or cholestatic conditions, conjugated bilirubin may be excreted via MRP3 (encoded by *ABCC3*) across sinusoidal membranes into blood, to a lesser extent, and reabsorbed by OATP1B1 and OATP1B3 [3, 16].

Lipids are also important components of bile. The heterodimeric transporter ABCG5/8 mediates cholesterol across canalicular membranes. Phosphatidylcholine (PC) is flopped by the floppase multidrug resistance P-glycoprotein 3 (MDR3, encoded by *ABCB4*) to the outer lipid leaflet and then extracted by bile salts into bile to form micelles. The combination of cholesterol and sphingomyelin makes membranes highly detergent resistant [17, 18]. The flippase FIC1(*ATP8B1*) is required to flip phosphatidylserine (PS) back from the outer lipid leaflet to the inner lipid leaflet of the canalicular membrane to stabilize the integrity of the canalicular membrane [19]. Additionally, FIC1 is required for the functional expression of MDR3 [20]. Thus, hepatocytes and biliary epithelium are protected from bile acid toxicity through the efflux of bile acids mediated by BSEP and the functions of MDR3 and FIC1.

### Homeostasis of bile acid pools

The homeostasis of bile acids is tightly controlled by the de novo synthesis of bile acids and the expression of transporters that affect hepatocellular bile acid levels. The key regulating molecules are farnesoid X receptor (FXR, *NR1H4*) and membrane-bound Takeda G protein-coupled receptor (TGR5) [6]. FXR is a nuclear receptor that is highly expressed in hepatocytes and enterocytes in the distal small intestine and colon. TGR5 is expressed in enteroendocrine cells, gallbladder cells and cholangiocytes. FXR forms heterodimers with other nuclear receptors to mediate its transcriptional activity [21–24]. Upon binding with bile acids as its natural ligands, FXR downregulates the expression of bile acid synthesis enzymes (mainly CYP7A1) and the sinusoidal uptake transporter of NTCP but upregulates the expression of the bile acid efflux transporter BSEP to reduce intracellular bile acid concentrations [25–29]. When bile acids are accumulated in hepatocytes, activated hepatic FXR increases sinusoidal bile acid efflux via MRP4 and heterodimeric OSTα/β [30, 31]. FXR also inhibits the expression of the ileal bile acid transporter ASBT to reduce the enterohepatic circulation of bile acids [32, 33]. Activation of FXR induces enterocytes to release FGF19. Through enterohepatic circulation via the portal vein, FGF19 translocates to the liver and inhibits the expression of CYP7A1 in the hepatocytes [34]. Through FXR, bile is controlled via a negative feedback loop at the transcriptional level via transporters and bile acid synthesis systems.

### Cholestasis

**Fig. 3:**
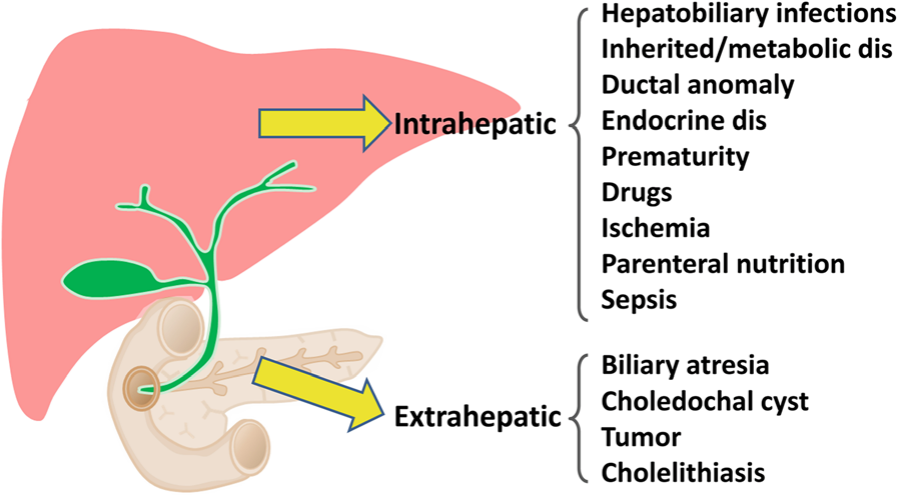
Etiologies of intrahepatic and extrahepatic cholestasis of inherited or secondary causes. dis: disorders

Cholestasis is defined as disturbances in bile flow caused by diseases either in the hepatocytes, intrahepatic bile ducts or extrahepatic biliary system. Cholestatic liver disease is one of the most common forms of liver disorders resulting from inherited or acquired liver diseases. Inadequate bile flow of any causes results in accumulation of bile contents, including bilirubin, bile acids, and lipids in the liver, and consequently cause elevated levels of bilirubin and bile salts in the liver and blood, as well as dysregulated lipid metabolisms. Clinically, patients usually manifest jaundice as a result of hyperbilirubinemia. Other symptoms include clay stool, pruritus, or infrequently, bleeding episodes such as intracranial hemorrhage. Chronic cholestatic liver disease may progress to liver cirrhosis and liver failure and is the leading cause of pediatric liver transplantation. According to the anatomical location of its occurrence, cholestasis is divided into extrahepatic and intrahepatic cholestasis. Extrahepatic cholestasis is caused by structural abnormalities of the biliary tract including the obstruction of bile ducts and the gallbladder. Surgical treatments are typically applied to restore the physiological function. However, intrahepatic cholestasis is more complicated and typically requires sophisticated investigations. The common causes of extrahepatic and intrahepatic cholestasis are shown in Fig. 3.

### Etiologies of inherited bilirubin metabolism disorders causing indirect hyperbilirubinemia

Disturbances in the bilirubin metabolisms result in accumulation of bilirubin in the liver and blood, and consequently cause hyperbilirubinemia detected by routine serum biochemistry test, or called jaundice clinically. Gilbert syndrome is a benign clinical condition usually present mild intermittent jaundice in children or adult. TA repeat polymorphism (UGT1A1*28) in the promoter of UGT1A1 gene is the most commonly affected region. Gilbert syndrome can be identified in the general population, and many are identified by blood test of a health exam [35].

Crigler-Najjar syndrome is also cause by mutations in the *UGT1A1* gene. Type I is a rare autosomal recessive disorder with complete loss of enzymatic function that cause extremely high bilirubin levels (above 20 mg/dL) and may lead to encephalopathy due to kernicterus. Treatments include phototherapy, exchange transfusion, or liver transplantation. Crigler-Najjar syndrome Type II manifests medium levels of hyperbilirubinemia (around 5–20 mg/dL), with retention of some enzymatic activity. Phenobarbital can be used intermittently to reduce bilirubin levels below 10-15 mg/dL.

Genetic variations in the *UGT1A1* gene, especially 211 G to A (G71R in exon 1) mutation, as well as variations in the glucose-6-phosphate dehydrogenase (*G6PD*) and *OATP2* genes, also contribute to the occurrence of neonatal jaundice and breast-feeding jaundice [36–38]. Homozygous 211 G to A mutation has been reported to be associated with severe neonatal jaundice.

### Etiologies of inherited cholestasis causing direct hyperbilirubinemia

**Fig. 4:**
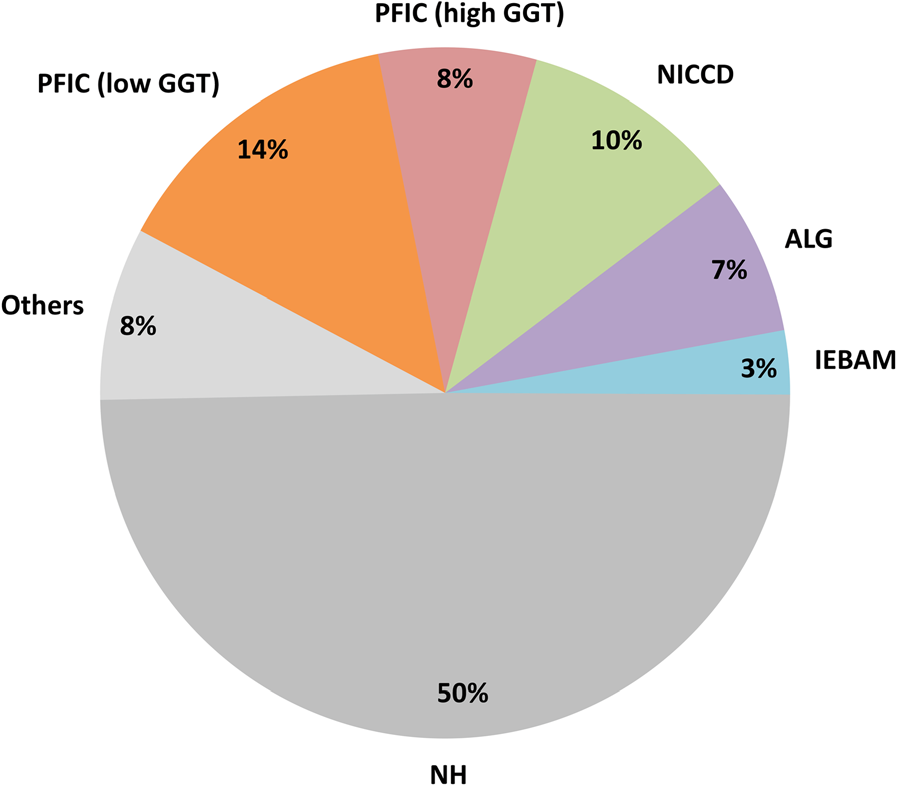
Distributions of final diagnosis of intrahepatic cholestasis in infancy in 135 Taiwanese infants 2000–2012. (Adapted from Lu FT et al., J Pediatr Gastroenterol Nutr 2014;59: 695–701). ALG, Alagille syndrome; GGT, gamma-glutamyl transpeptidase; IEBAM, inborn error of bile acid metabolism; NH, neonatal hepatitis; NICCD, neonatal intrahepatic cholestasis caused by citrin deficiency; PFIC, progressive familial intrahepatic cholestasis

Inherited cholestatic liver diseases may manifest early in life. The presenting age ranges from infancy to young adulthood. In the last 20 years, there has been tremendous progress in understanding the genetic background of cholestatic liver disease [39–43]. Table 1 lists the categories and genes involved in inherited genetic disorders. Up to now, more than 100 inherited diseases are identified to cause cholestatic liver diseases with the initial presentation of jaundice. Some disorders may be associated with congenital anomalies or with multiple organ involvement. We have previously investigated the genetic background of pediatric patients in Taiwan with BSEP, FIC1, MDR3 defects [44–47]. We have also reported adaptive changes of hepatocyte transporters associated with obstructive cholestasis in biliary atresia, an important extrahepatic cholestatic liver disease with common symptom of prolonged neonatal jaundice [48, 49]. The distribution of disease types in Taiwanese infants with intrahepatic cholestatic liver diseases is shown in Fig. 4.

**Table 1:** Differential diagnosis of jaundice caused by primary or secondary intrahepatic liver diseases

Diseases/phenotype	Gene (Alias)
*Indirect hyperbilirubinemia*	
Crigler-Najjar syndrome	UGT1A1
Gilbert syndrome	UGT1A1
*Direct hyperbilirubinemia*	
Progressive familial intrahepatic cholestasis	
PFIC1	ATP8B1 (FIC1)
PFIC2	ABCB11 (BSEP)
PFIC3	ABCB4 (MDR3)
Others	
	TJP2 (ZO2)
	NR1H4 (FXR)
	Myosin 5B (MYO5B)
Bilirubin Transport Defects	
Rotor syndrome	SLCO1B1 (OATP1B1)/ SLCO1B3 (OATP1B3)
Dubin-Johnson syndrome	ABCC2 (MRP2)
Syndromic cholestasis	
Alagille syndrome (paucity of interlobular bile ducts)	JAG1
	NOTCH2
Arthrogryposis-renal dysfunction-cholestasis syndrome.	VPS33B
	VIPAR
Inborn errors of bile acid metabolisms	
Bile acid synthetic defects	HSD3B7
	AKR1D1 (SRD5B1)
	CYP7B1
Bile acid conjugation defects	BAAT
Cerebrotendinous Xanthomatosis	CYP27A1
Metabolic liver disease	
Wilson disease	ATP7B
Alpha-1-antitrypsin deficiency	SERPINA1
Cystic fibrosis	CFTR
Neonatal cholestasis caused by citrin deficiency (type 2 citrullinemia)	SLC25A13 (CITRIN)
Niemann-Pick disease type C (NPC)	NPC1
	NPC2
Wolman disease	LIPA
Hepatic mitochondriopathy	
	TWNK (C10orf2), DGUOK, MPV17, POLG, BCS1L, RRM2B, SCO1, SUCLG1
Neonatal sclerosing cholangitis	
	CLDN1
Polycystic diseases (polycystic kidney disease; polycystic liver diseases; ductal plate malformation)	
	PKD1, PKD2, PRKCSH, SEC63, PKHD1
Diseases with multi-organ involvement	
Down syndrome	
Endocrine disorders	
Hypopituitarism	
Hypothyroidism	
Hemophagocytic lymphohistiocytosis (HLH)	
Infections	
Viral infections (cytomegalovirus, enterovirus, EB virus, HIV, etc.)	
Bacteria infection, sepsis	
Toxoplasma	
Ischemia	
Shock, heart failure, cardiovascular surgery	
Parenteral nutrition-associated cholestasis	
Drugs	
Toxins

Progressive familial intrahepatic cholestasis (PFIC) is a clinical syndrome with features of chronic intrahepatic cholestasis that typically begin in infancy and progress to biliary cirrhosis and hepatic failure by the first or second decade of life [40, 46, 50]. The first three types of genetic defects identified are commonly referred to as PFIC1, PFIC2, and PFIC3. PFIC1 and PFIC2 are characterized by low serum γ-glutamyltransferase (GGT) levels. PFIC1 (Byler’s disease) patients have FIC1 gene mutations, and PFIC2 patients have mutated BSEP gene. PFIC3 is characterized by high serum GGT levels and is caused by genetic mutations in the MDR3 gene [51, 52]. BSEP plays a pivotal role in bile physiology as it mediates canalicular bile salt export and is the main driving force of bile flow [53].

With the advances in genetic technologies in recent years, novel disease-causing genes for PFIC have been reported. FXR, the key regulator of bile acid metabolism, have been implicated in a novel form of infant cholestasis with liver failure in two European families [54]. We also identified a fatal case of infant cholestasis with liver failure occurring before 3 months of age [55]. Additionally, TJP2 and MYO5B have been found to cause PFIC. TJP2 is an important component of tight junctions, and a deficiency of TJP2 disrupts the tight-junction structure in the liver [56]. MYO5B is associated with low GGT infant cholestasis. MYO5B is an actin-based motor protein and an effector of Rab11a/b. MYO5B mutations result in the dysregulation of Rab proteins and further disrupt the trafficking of BSEP [57, 58]. Doublecortin domain containing 2 (DCDC2), a tubulin-binding protein, is associated with renal-hepatic ciliopathy and neonatal sclerosing cholangitis [59–61]. The mitochondrial transcription factor TFAM is associated with mitochondrial DNA depletion syndrome [62]. Recently, a homozygous single nucleotide deletion in organic solute transporter-β (OSTβ/SLC51B) was demonstrated to cause congenital diarrhea and cholestasis [63].

Dubin-Johnson and Rotor syndrome are two inherited disorders manifesting direct hyperbilirubinemia but with normal or minimally elevated alanine transaminase (ALT) levels, clinically manifesting as jaundice. Dubin-Johnson syndrome is caused by disruption of MRP2 and characterized by grossly black livers and pigment deposition in hepatocytes. Neonatal cholestasis caused by Dubin-Johnson syndrome has been reported in Taiwan and Japan [64, 65]. Our group has identified patients recovered from neonatal cholestasis had re-emergence of jaundice in young adulthood after long-term follow-up [64]. Rotor syndrome has recently been identified to be caused by genetic disruption of both *SLCO1B1* and *SLCO1B3* genes [66, 67]. These two disorders are benign and do not require specific treatment.

Genetic cholestasis not only causes pediatric liver disease but may also be present in adult liver disease. Additionally, adult liver diseases may result from genetic liver diseases. In general, protein functional disturbances are less detrimental and are typically caused by missense genetic mutations or multifactorial disorders. Cholestasis in pregnancy has been associated with genetic variants/mutations in *ABCB4*, *ABCB11*, *ATP8B1*, *ABCC2* and *TJP2* [68]. Adult benign recurrent intrahepatic cholestasis (BRIC) is also associated with PFIC-related genes and may have mutations that are less damaging [69–72]. Acquired forms of cholestasis, such as drug-induced liver disease, have also been associated with genetic variants [73, 74].

Diseases related to ductal plate malformation are an important group of developmental disorders that lead to a paucity or malformation of intrahepatic or interlobular bile ducts. Alagille syndrome, first described by Alagille et al., is based on clinical diagnostic criteria including a characteristic face; a paucity of interlobular bile ducts in liver pathology; and cardiac, eye, and vertebral anomalies [75]. The *JAG1* mutation accounts for > 90% of cases of Alagille syndrome, and mutations in *NOTCH2* have been described in a minority of patients [76]. Other syndromic disorders and polycystic liver/kidney diseases may also present with infant cholestasis as the first symptom.

Cholestasis is a common manifestation of hepatic metabolic disorders, including carbohydrate, amino acid, and fat metabolism, as well as mitochondrial and endocrine anomalies. Most of these diseases are rare disorders, and the disease incidence largely depends on ethnic background. For example, neonatal cholestasis caused by citrin deficiency (NICCD) is an important cause of cholestasis in East Asian children [77, 78]. We have previously identified facial features and biochemical characteristics for the phenotypic diagnosis of NICCD [79, 80]. Alpha 1-antitrypsin (A1AT/SERPINA1) deficiency and cystic fibrosis are important causes in western countries but how lower incidences in Asian populations.

Inborn errors of bile acid metabolism constitute a group of important metabolic disorders causing infant cholestasis. Notably, oral primary bile acid supplementation is effective and can avoid patient deterioration and the need for liver transplantation upon timely treatment [81, 82].

Neonatal hemochromatosis is an important cause of neonatal liver failure that manifests as early onset cholestasis. However, recent studies have elucidated this condition as a disorder of gestational alloimmune liver diseases instead of hereditary hemochromatosis [83]. Treatment involves exchange blood transfusion and intravenous immunoglobulin applied as early as when the neonate is born.

Other congenital anomalies, such as chromosomal anomalies, endocrine disorders, and developmental disorders may also cause cholestasis. Liver disease is typically a multi-organ manifestation of congenital anomalies.

### Diagnosis

#### Clinical history

A careful clinical history is important to investigate common secondary causes of jaundice and cholestasis, including hemolytic anemia, G6PD deficiency, hereditary spherocytosis and other red cell membrane disorders, prematurity, sepsis, drug-induced liver injury, parenteral nutrition-associated liver diseases, ischemia, and pregnancy. Ethnic background and parental consanguinity are clues for certain types of inherited liver disorders.

#### Phenotypic diagnosis

The traditional phenotypic diagnosis includes low GGT as a signature of PFIC1 (FIC1 defect) and PFIC2 (BSEP defect). GGT levels, Byler’s bile in electron microscopy, and duodenal biliary bile content can be used as clinical markers to indicate further genetic confirmation [84, 85]. Syndromic cholestasis, including Alagille syndrome, can be diagnosed by phenotypic criteria [75]. Patients with NICCD have phenotypic features, and we have developed a clinical scoring system to aid in diagnosis [79, 86]. Importantly, investigating the involvement of extrahepatic organs is important for differential diagnosis.

#### Biochemical diagnosis

For patients suspecting jaundice or cholestasis, routine liver biochemistry tests include total and direct bilirubin levels, aspartate transferase levels, ALT levels, GGT and alkaline phosphatase (ALP) levels. Low serum GGT level disproportionate to severity of cholestasis is a clinical clue for inherited cholestasis such as PFIC and inborn errors of bile acid synthesis. Some disorders with metabolic signatures can be diagnosed with biochemical analysis. Diseases, such as inborn error of bile acid metabolism (IEBAM), [87] and metabolic disorders, such as NICCD, [86] require analysis by mass spectrometry.

#### Genetic diagnosis

Genetic diagnosis is a definitive diagnosis for inherited genetic liver diseases, as many of these diseases lack adequate biomarkers. Genetic tests have largely evolved in the past two decades due to the tremendous progress of genetic analysis technologies. Conventional genetic diagnosis uses direct sequencing for selected genes based on the phenotype of the patient. High-throughput methods have subsequently been developed, such as a resequencing chip that detects 5 genes for genetic cholestasis (*SERPINA1*, *JAG1*, *ATP8B1*, *ABCB11*, and *ABCB4*) in 2007 [88]. Denaturing high-performance liquid chromatography and high-resolution melting analysis have been used to detect single-gene variants in large numbers of patients [46, 79]. Recent next generation sequencing (NGS) panels in liver diseases have incorporated a limited number of genes, particularly PFIC [65, 89]. Expanded panel-based NGS involving more than 50 genes has been used in clinical patients with promising results [55, 90]. Whole exome sequencing has been applied to identify novel disease-causing genes [57, 63].

### Treatment

#### Nutritional support

Bile mediates the intestinal absorption of fat and fat-soluble vitamins. In cholestatic liver diseases, the defective absorption of fat and fat-soluble vitamins (vitamins A, D, E, and K) is commonly observed but clinically obscure. Fat malabsorption results in calorie insufficiency and failure to thrive, especially in early childhood. Patients are advised to use formulas containing medium-chain triglycerides or add oils containing medium-chain triglycerides to their food. Deficiency in fat-soluble vitamins may result in multiple organ dysfunctions, including rickets, coagulopathy, and defective neurological, immunological and visual functions. Without supplementation, symptoms of deficiency, such as coagulopathy, osteoporosis, fracture, growth failure and life-threatening hemorrhage, may occur in patients. In addition, deficiencies in fat-soluble vitamins may also cause inadequate anti-oxidation, which is frequently overlooked in clinical patients.

#### Medical treatment

Although jaundice is the common manifestation of the highly variable etiologies, treatment does not target only to jaundice improvement (to reduce serum bilirubin level), but to target the underlying disorders that may cause hepatobiliary injury and progressive fibrosis and cirrhosis, which is usually associated with elevated bile acid levels or abnormal metabolites. Additional treatment goals are to improve nutritional status, pruritus and life quality, to prevent or to treat cirrhosis related complications.

PFICs, Alagille syndrome, and inborn errors of bile acid synthesis are the most devastating disorders that cause cirrhosis and may need liver transplantation. Effective treatment options for PFICs and Alagille syndrome are limited. Several drugs are under investigation and clinical trial. Here we will discuss about the standard treatment and several newly developed therapeutic strategies for these disorders.

Ursodeoxycholic acid (UDCA) has widely been used to treat cholestatic liver disease and is effective to improve biochemical parameters and pruritus [91]. However, UDCA is not an ideal therapeutic option for PFIC2 patients with BSEP defects. In animal models, UDCA may aggravate liver injury due to the inability of BSEP to export UDCA from hepatocytes [92]. There is a need to develop new drugs targeting BSEP defects. Missense mutations in *BSEP/ABCB11* impair protein translation or intracellular trafficking, which reduce canalicular expression of BSEP and eventually cause cholestasis. Recent studies have indicated that 4-phenylbutyrate (4-PB, Buphenyl), a clinically approved pharmacological chaperone, can be used to restore the canalicular expression of BSEP. By using MDCK II cells and SD rats, Hayashi et al. reported that 4-PB significantly relocalizes and enhances the cell surface expression of both wild-type and mutated rat Bsep [93]. Besides its effect on Bsep expression, 4-PB treatment significantly increased hepatic MRP2 and decreased serum bilirubin level in patient with ornithine transcarbamylase deficiency (OTCD) [94]. Moreover, Gonzales et al. applied 4-PBA to PFIC2 patients and successfully restored the hepatic secretion of bile acids and decreased total serum bilirubin via the re-localization of mutated BSEP to canalicular membranes [95]. In addition to 4-PB, steroids are a therapeutic option to enhance BSEP expression. Cell culture experiments have suggested that dexamethasone upregulates Bsep and Mrp2 at the mRNA level in rat primary hepatocytes [96, 97], and treatment with glucocorticoids induces the expression of Bsep, Mrp2, and cytochrome P450 oxidase in rat livers [98]. Additional animal experiments and clinical tests have shown that steroid treatment improved bile homeostasis. For example, dogs receiving a high dosage of hydrocortisone (5 mg/kg) showed a significant increase in bile flow [99]. Engelmann et al. reported that steroids effectively ameliorated cholestatic itches and reduced the serum level of bile salts and bilirubin in two PFIC2 patients carrying missense mutations in BSEP [100].

Blocking enterohepatic circulation has been recently shown as a promising strategy to reduce the hepatic accumulation of bile acids in PFIC2 patients. After secretion from the gallbladder into the intestine, a majority of bile acid is absorbed by enterocytes via ASBT and recycled to liver via enterohepatic circulation. Two independent animal studies have shown that small molecule ASBT inhibitors, SC-425 and A4250, effectively reduced the enteric uptake of bile acid, decreased serum total bilirubin levels, and improved liver fibrosis and inflammation in Mdr2 knockout mice, an animal model of PFIC3 [101]. Moreover, on March 2018, A4250 successfully passed clinical phase II trials (ClinicalTrials.gov Identifier: NCT02630875).

The recently developed FXR agonist (Obeticholic acid) has been demonstrated to improve the ALP level in primary biliary cirrhosis [102], and has also been investigated for the treatment of nonalcoholic steatohepatitis (NASH) [103–105].

Certain types of the inborn errors of bile acid metabolism are treatable [81]. Oral cholic acid therapy is indicated for 3β-Hydroxy-Δ(5)-C27-steroid oxidoreductase (HSD3B7) deficiency, Δ (4)-3-oxosteroid 5β-reductase (SRD5B1, AKR1D1) deficiency, and Zellweger spectrum disorders [106]. CDCA has also been reported to be effective for oxysterol 7α-hydroxylase (CYP7B1) deficiency, cerebrotendinous xanthomatosis, and other forms of bile acid synthetic defects [107]. After treatment, patients may recover from liver dysfunction, free of jaundice, and avoid liver transplantation. Life-long therapy is indicated for the oral supplementation. Early diagnosis and treatment is important to improve outcome.

Many patients with cholestatic liver disease suffer from pruritus, except patients with inborn errors of bile acid synthesis. Alagille syndrome, PFIC1 and 2 commonly cause disturbing pruritus, which affects daily life quality. Antihistamines, rifampin, and cholestyramine have been used to partially improve the symptoms of this condition. UV-B phototherapy is an alternative therapy to treat pruritus.

### Biliary diversion and nasogastric drainage

Palliative treatment with biliary diversion surgery by the disruption of enterohepatic circulation may relieve pruritus and improve liver biochemical profiles. Several strategies have been used, including external biliary diversion or ileal exclusion [108–110].

### Liver transplantation

Liver transplantation is considered a curative treatment for various liver diseases [111]. However, for PFIC2 patients, the recurrence of the BSEP defect has been reported due to circulating BSEP antibodies [112, 113]. Anti-CD20 antibody and plasmapheresis have been reported to treat recurrent BSEP deficiency [114]. The outcomes in BSEP defects of common European mutations, such as D482G, are better than those of other mutation types [85]. In addition, patients with multi-organ manifestations, such as diarrhea and pancreatic insufficiency in PFIC1, cannot be treated by liver transplantation.

### Liver tumor surveillance

The disruption of bile acid transport not only causes PFIC but has also been associated with hepatocellular carcinoma and cholangiocarcinoma [115, 116]. Patients with BSEP deficiency and tyrosinemia are of greater risk of developing hepatocellular carcinoma (HCC). It is mandatory that patients with PFIC be screened for liver tumors on a regular basis. Alpha-fetoprotein is not typically elevated. Some patients were found to have HCC in the explanted liver. Thirty-eight out of 175 pediatric HCC patients receiving liver transplantation were diagnosed with inherited liver diseases [117].

### Hepatocyte transplantation and gene therapy

Liver transplantation is often an ultimate option for patients with severe cholestasis, but the rarity of organ sources is an important issue. Hepatocyte transplantation might be an alternative therapy to use efficiently donor tissue in a less invasive manner. Cell therapy has been investigated in animal models with various extents of hepatocyte repopulation, including models of PFIC3 (*Mdr2* knockout mice), PFIC2 (*Abcb11* knockout mice) and hereditary tyrosinemia [118–120]. In previous studies, we found that UDCA can provide a selective growth advantage to donor hepatocytes in *Abcb11* knockout mice and enhance the repopulation of donor hepatocytes and partially correct the bile acid profile [92]. However, insufficient long-term substitution ratio of donor hepatocyte in the livers of recipients, and the lack of donor cell sources limits the wide application of UDCA to treat clinical patients. For the past two decades, more than 20 patients with inherited liver-based metabolic disorders have received hepatocyte transplantation. Most of these patients showed only partial and transient improvements in metabolic function for several months and finally underwent liver transplantation [121–123]. Among these individuals, two patients with PFIC2 showed no obvious benefits after hepatocyte transplantation, as the existing fibrosis impaired the engraftment of the transplanted hepatocytes [122]. Recently, glyceryl trinitrate have been shown to enhance the efficacy of the transplanted hepatocyte repopulation in *Mdr2* knockout mice [124]. With additional treatment to boost donor cell repopulation, hepatocyte transplantation might be refined and benefit patients with cholestasis.

Few studies on experimental gene therapy for cholestatic liver diseases have been reported. The adenoviral transfer of the aquaporin-1 gene has been shown to improve bile flow in rats with estrogen-induced cholestasis, but the effect in inherited cholestatic disease has not been validated [125].

## Conclusions

With the revolutionary development of genetic analysis technologies, we have largely elucidated the molecular mechanisms of jaundice, bile flow and bile metabolism and identified new causes of genetic liver diseases that cause cholestasis. The understanding of “bile biology” not only provides insights into the mechanisms of liver pathophysiology but also facilitates the diagnosis of genetic liver diseases and the development of novel treatments.

## Abbreviations

4-PB: 4-phenylbutyrate
A1AT: Alpha 1-antitrypsin
ABC: ATP-binding cassette
AFP: Alpha-fetoprotein
ASBT: Apical sodium dependent bile acid transporter
BAs: Bile acids
BSEP: Bile salt export pump
CA: Cholic acid
CDCD: Chenodeoxycholic acid
CYP: Cytochrome P450
DCA: Deoxycholic acid
DCDC2: Doublecortin domain containing 2
FGFR4: fibroblast growth factor receptor 4
FXR: farnesoid X receptor
G(T)CA: Glyco- or tauro-cholic acid
G(T)CDCA: Glyco- or tauro-chenodeoxy cholic acid
GGT: Gamma glutamyl transpeptidase
HCC: Hepatocellular carcinoma
IEBAM: Inborn error of bile acid metabolism
LCA: Lithocholic acid
MDR2/3: Multidrug resistance protein 2/3
MRP2/3/4: Multidrug resistance-associated protein 2/3/4
MYO5B: Myosin VB
NASH: Nonalcoholic steatohepatitis
NH: Neonatal hepatitis
NICCD: Neonatal cholestasis caused by citrin deficiency
NTCP: Sodium/taurocholate co-transporting polypeptide
OATP1B1/3: Organic-anion-transporting polypeptide 1B1 and 1B3
OSTα/β: Organic solute transporter-α/β
PC: Phosphatidylcholine
PFIC: Progressive familial intrahepatic cholestasis
PS: Phosphatidylserine
RXRα: Retinoid X receptor α
SERPINA1: Serpin family A member 1
SHP: Small heterodimer partner
TFAM: Mitochondrial transcription factor A
TGR5: Takeda G protein-coupled receptor 5 (G protein-coupled bile acid receptor 1 , GPBAR1)
TJP2: Tight junction protein 2
UDCA: Ursodeoxycholic acid
UGT1A1: UDP-glucuronosyltransferase 1A1
